# Burden, Antibiotic Resistance, and Clonality of *Shigella* spp. Implicated in Community-Acquired Acute Diarrhoea in Lilongwe, Malawi

**DOI:** 10.3390/tropicalmed6020063

**Published:** 2021-04-28

**Authors:** Abel F.N.D. Phiri, Akebe Luther King Abia, Daniel Gyamfi Amoako, Rajab Mkakosya, Arnfinn Sundsfjord, Sabiha Y. Essack, Gunnar Skov Simonsen

**Affiliations:** 1 Antimicrobial Research Unit, College of Health Sciences, University of KwaZulu-Natal, Durban 4000, South Africa; abelphiri19@yahoo.com (A.F.N.D.P.); amoakodg@gmail.com (D.G.A.); essacks@ukzn.ac.za (S.Y.E.); 2 National Microbiology Reference Laboratory, Ministry of Health, Lilongwe 3, Malawi; 3 Department of Pathology, College of Medicine, University of Malawi, Blantyre 3, Malawi; rmkakosya@medcol.mw; 4 Department of Microbiology and Infection Control, University Hospital of North Norway, 9038 Tromsø, Norway; arnfinn.sundsfjord@uit.no (A.S.); gunnar.skov.simonsen@unn.no (G.S.S.); 5 Faculty of Health Sciences, UiT—The Arctic University of Norway, N-9037 Tromsø, Norway

**Keywords:** Diarrhoeal diseases, antibiotic resistance, public health, low- and middle-income countries, clonal relatedness, virulence determinants, *Shigella* species, molecular epidemiology

## Abstract

Although numerous studies have investigated diarrhoea aetiology in many sub-Saharan African countries, recent data on *Shigella* species’ involvement in community-acquired acute diarrhoea (CA-AD) in Malawi are scarce. This study investigated the incidence, antibiotic susceptibility profile, genotypic characteristics, and clonal relationships of *Shigella flexneri* among 243 patients presenting with acute diarrhoea at a District Hospital in Lilongwe, Malawi. *Shigella* spp. were isolated and identified using standard microbiological and serological methods and confirmed by identifying the *ipaH* gene using real-time polymerase chain reaction. The isolates’ antibiotic susceptibility to 20 antibiotics was determined using the VITEK 2 system according to EUCAST guidelines. Genes conferring resistance to sulfamethoxazole (*sul1*, *sul2* and *sul3*), trimethoprim (*dfrA1*, *dfrA12* and *dfrA17*) and ampicillin (*oxa-1* and *oxa-2*), and virulence genes (*ipaBCD*, *sat*, *ial*, *virA*, *sen*, *set1A* and *set1B*) were detected by real-time PCR. Clonal relatedness was assessed using ERIC-PCR. Thirty-four *Shigella flexneri* isolates were isolated (an overall incidence of 14.0%). All the isolates were fully resistant to sulfamethoxazole/trimethoprim (100%) and ampicillin (100%) but susceptible to the other antibiotics tested. The *sul1* (79%), *sul2* (79%), *sul3* (47%), *dfrA12* (71%) and *dfrA17* (56%) sulfonamide and trimethoprim resistance genes were identified; *Oxa-1*, *oxa-2* and *dfrA1* were not detected. The virulence genes *ipaBCD* (85%), *sat* (85%), *ial* (82%), *virA* (76%), *sen* (71%), *stx* (71%), *set1A* (26%) and *set1B* (18%) were detected. ERIC-PCR profiling revealed that the *Shigella* isolates were genetically distinct and clonally unrelated, indicating the potential involvement of genetically distinct *S. flexneri* in CA-AD in Malawi. The high percentage resistance to ampicillin and sulfamethoxazole/trimethoprim and the presence of several virulence determinants in these isolates emphasises a need for continuous molecular surveillance studies to inform preventive measures and management of *Shigella*-associated diarrhoeal infections in Malawi.

## 1. Introduction

The global burden due to diarrhoeal disease was estimated at close to 700 million in 2015 with an associated death of almost 500 thousand, as reviewed by Kotloff et al. [1]. One of such diseases is shigellosis, which is feared to become more deadly in the future [2]. Shigellosis is an acute invasive enteric infection often clinically manifested as bloody diarrhoea [3] and is among the top 1% causes of diarrhoeal diseases-associated deaths globally, especially in children under the age of 5 years [4]. This infection is caused by members of the genus *Shigella*, which are small, non-motile Gram-negative rod-shaped bacteria within the *Enterobacterales* family [5]. There are four species of *Shigella* classified based on biochemical reactions, serological differences and genetic relatedness, i.e., *S*. *dysenteriae*, *S. flexneri*, *S. boydii* and *S*. *sonnei* [6]. *S. dysenteriae, S. flexneri* and *S. boydii* are the most prevalent in developing countries [7,8]. Despite a decline in global mortality due to this infection over the past 25 years, the disease is still a major cause of deaths in children <5 years of age, especially in low-income countries, contributing to up to 21% of deaths [9].

Fluoroquinolones, cephalosporins and sulfonamides are the drugs of choice for the clinical management of acute diarrhoea caused by *Shigella* spp. [10], and are included in the Malawi Standard Treatment Guidelines [11]. However, these bacteria are increasingly becoming resistant to these drugs, using various antibiotic resistance mechanisms, and limiting treatment options of *Shigella* infections [12,13,14]. Mutations in *gyrA* and *parC* genes are responsible for resistance to fluoroquinolones in *Shigella* spp. [15]. β-lactamases, capable of hydrolysing penicillins, first-, second- and third-generation cephalosporin are the most frequently encountered resistance mechanisms to β-lactam antibiotics [16], with OXA β-lactamases implicated in ampicillin resistance [17]. Sulfonamide resistance is most frequently caused by a genetic alteration in the chromosomal dihydropteroate synthase gene (*folP*) or the acquisition of *sul*-type determinants encoding enzymes with lower sulfonamide binding ability [18]. Three different variants of *sul*-type resistance genes have been described in the literature, viz., *sul1*, *sul2* and *sul3*. *Sul1* is commonly found in association with class 1 integrons [19], whereas *sul2* genes are usually located on various plasmids. *Sul3* has several enzyme variants [20]. All these genetic determinants exist in *Enterobacterales* recovered from human samples, including stool. The increased resistance of these organisms to currently used antibiotics, has even prompted a drastic search towards the development of vaccines to prevent the spread of infection [21]. However, while these solutions are being sought, there is need for more characterisation of *Shigella* species, especially in impoverished communities.

Although several studies have investigated the incidence and antibiotic resistance of *Shigella* species in low-income Sub-Saharan Africa and South Asia countries [22], these organisms continue to cause significant loss of lives in these regions, and recent data on clinical isolates from most of these countries, including Malawi, are lacking. Such paucity in current data could have severe public health consequences in these regions, especially given their substandard sanitary conditions and weak healthcare systems. Thus, this study investigated the antibiotic susceptibility profile, resistance mechanisms, virulence factors and clonal relationship among clinical *Shigella* spp. isolated from patients with acute diarrhoea in a community setting in Lilongwe, Malawi.

## 2. Materials and Methods

### 2.1. Ethical Consideration

Ethical approval was granted by the Biomedical Research Ethics Committee (BREC) of the University of KwaZulu-Natal (Reference number: 203/15) and the College of Medicine Research Ethics Committee (COMREC) of the University of Malawi (Reference number: P.05/15/1740). Voluntary informed consent was obtained from the study participants and guardians of minors as applicable.

### 2.2. Study Design, Sample Population and Sample Collection

This was a prospective, observational study where stool samples were collected upon explicit, voluntary informed consent from patients clinically diagnosed with acute diarrhoea. Patients were recruited based on the clinical diagnosis of acute diarrhoea by clinicians following routine procedures at the Outpatient Department (OPD) of Bwaila Hospital in Lilongwe, Malawi, over six months from November 2015 to May 2016. After explaining the study’s purpose and obtaining informed consent, patients were given wide-mouth plastic sterile containers with lids for stool sample collection. The samples were immediately transported to the laboratory on ice for analysis.

### 2.3. Culture and Identification of Isolates

Stool samples were directly plated, using sterile disposable inoculation loops, on MacConkey and Xylose Lysine Deoxycholate (XLD) agar (Beckton & Dickinson, Franklin Lakes, NJ, USA), and incubated aerobically at 35 °C for 18 to 24 h. Identification of *Shigella* was confirmed by conventional biochemical tests. Colonies suspected to be *Shigella* (small pale colonies on MacConkey and red colonies on XLD) were stabbed in Triple Sugar Iron Agar (TSI), Sulphur Indole Motility Agar (SIM) and Lysine Decarboxylase Citrate Agar (LDC) (Beckton & Dickinson, NJ, USA), and subjected to the urease production test. *Shigella sonnei* ATCC 9290 served as the control. Presumptive *Shigella* isolates were serologically grouped by slide agglutination tests using commercial *Shigella* polyvalent antisera (Beckton & Dickinson, NJ, USA) against the four *Shigella* species.

### 2.4. Molecular Confirmation of Shigella spp.

All presumptive *Shigella* isolates were confirmed by real-time PCR, targeting the *ipaH* gene [23]. DNA was extracted using the GeneJET Genomic DNA Purification kit according to the manufacturer’s instructions (Thermo Fisher Scientific, Waltham, MA, USA). The extracted DNA was used as a template for real-time PCR. The PCR assays were performed in total volumes of 10 µL: 5 µL of Luna^®^ universal qPCR master mix (New England Biolabs, Ipswich, MA, USA), 0.5 µL primers (forward and reverse, final concentration 0.5 µM), 3 µL template DNA and 1 µL of nuclease-free water.

After an initial enzyme activation at 98 °C for 50 s (hot start), the following cycling conditions were used: denaturation at 95 °C (10 s), annealing at 62 °C (30 s) and extension at 72 °C (20 s) for 35 cycles. A final extension at 72 °C for 5 min was followed by melt-curve analysis involving ramping up the melting temperature from 60 °C to 95 °C at a ramp rate of 0.15 °C at each step on a continuous mode. The melt-curve analysis was carried out using the QuantStudio Design and Analysis Software, Version 1.4.2.

All PCR constituents were purchased from Inqaba Biotechnical Industries (Pty) Ltd., Pretoria, South Africa. Each assay included a reaction mixture without DNA as a No Template Control and DNA from *Shigella sonnei* ATCC 9290 as a positive control. The reactions were run on a QuantStudio^®^ 5 Real-Time PCR System (Thermo Fisher Scientific, Waltham, MA, USA).

### 2.5. Antibiotic Susceptibility Testing (AST)

Antibiotic susceptibility testing was done using the VITEK2 system according to the manufacturer’s recommendations (bioMerieux, Marcy-l’Étoile, France) with the antibiotic panel consisting of ampicillin, amoxicillin-clavulanic acid, piperacillin-tazobactam, cefoxitin, cefuroxime, cefotaxime, ceftazidime, cefepime, cotrimoxazole, imipenem, meropenem, ertapenem, doripenem, gentamicin, amikacin, ciprofloxacin, levofloxacin, moxifloxacin, sulfamethoxazole/trimethoprim and tigecycline. Results were interpreted according to EUCAST guidelines [24]. *E. coli* ATCC2592 was used for quality control.

### 2.6. Identification of Resistance and Virulence Genes

Isolates were examined for the presence of resistance genes (sul1, sul2 [25], sul3 [20], dfrA1 [26], dfrA12 [27], dfrA17 [28], oxa-1 [29] and oxa-2 [30]) and Shigella-associated virulence genes (ipaBCD, sat, ial, virA, sen, stx, set1A, set1B) [31]

The PCR for *sul1*, *sul2* and *sul3* were performed using gene-specific primers (Table S1) in reaction volumes and cycling conditions as previously described for the isolates’ molecular confirmation. The remaining resistance genes were identified through conventional PCR, as previously described [26,27,28,29,30] (Table S1).

The cycling conditions for identifying the virulence genes were as previously described [31] but included a melt-curve analysis after the final extension. Unlike the *sul1, sul2*, *sul3* and *ipaH* genes, ramping for the virulence genes was done from 60 °C to 94 °C. *Shigella sonnei* ATCC 9290 was used as the control. Primers and annealing temperatures for amplifying the virulence genes are shown in Table S2.

### 2.7. Analysis of Clonality Using Enterobacterial-Repetitive-Polymerase Chain Reaction (ERIC-PCR)

ERIC-PCR analysis was carried out to determine the genetic relationship between the isolates. Previously extracted genomic DNA (2 µL) was in a total reaction volume of 10 µL containing 0.1 µL (10 µM) primers and 5 µL DreamTaq, and the volume made up with nuclease-free water. The primers ERIC_1 5′-ATG TAA GCT CCT GGG GAT TCA C-3′ and ERIC_2 5′-AAG TAA GTG ACT GGG GTG AGC G-3′ were used [32], and the PCR conditions were as previously described by Chukwu et al. [33]. ERIC-PCR products were loaded on a 1% (w/vol) agarose gel and visualised by UV transillumination (Syngene, Cambridge, UK) after staining in 0.1 mg/mL ethidium bromide for 15 min. Genotypic variation was analysed using the Gel CompareII version 6.0 software package (Applied Maths, Sint Martens-Latem, Belgium) by Unweighted Pair Group Method with Arithmetic mean (UPGMA) cluster analysis to produce a dendrogram. A similarity cut-off of 70% was used to define clonal clusters or ERIC types.

## 3. Results

### 3.1. Prevalence of Shigella spp. and Patients’ Demographics

A total of 243 faecal samples collected from out-patients clinically diagnosed with acute diarrhoea were tested for the presence of *Shigella* spp. from November 2015 to May 2016. Patients’ ages ranged from 10 to >69 years. A high proportion of samples was retrieved from males (*n* = 223; 91.7%) as opposed to females (*n* = 20, 8.2%). The predominant age group in males was from 12–42 years, while in females, it was from 16–53 years.

In this case, 34 stool samples were positive for *Shigella*, all of which were identified as *Shigella flexneri*, representing an overall incidence of 14.0%. Of these, four were females (16–37 years) while 30 (12–45 years) were males. Patients were from 14 different locations within the Lilongwe community, with the Chinsapo area recording the highest number of *S. flexneri* isolates (*n* = 7), followed by Area 36 (*n* = 6).

### 3.2. Antibiogram and Analysis of Resistance Genes

All 34 isolates were resistant to ampicillin and sulfamethoxazole/trimethoprim. One or more sulfonamide *sul* resistance genes were detected in 29/34 (85%) of the isolates, with *sul1* (27/34; 79%) and *sul2* (27/34; 79%) being more prevalent than *sul3* (16/34; 47%). Fifteen isolates (44%) harboured all three *sul* determinants. *Oxa-1* and *oxa-2* genes were not detected in any of the isolates. Trimethoprim resistance genes *dfrA12* (24/34; 71%) and *dfrA17* (19/34: 55%) were detected, whereas no isolates harboured the *dfrA1* gene (Table S3). All isolates were susceptible to the other antibiotics tested.

### 3.3. Detection of Genes Encoding Virulence Factors

**Figure 1:**
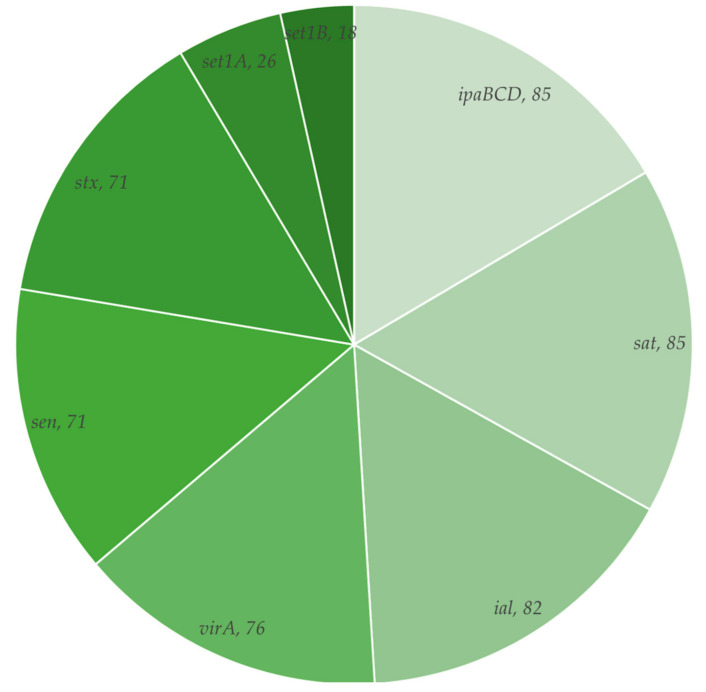
Distribution of virulence genes in *Shigella flexneri* isolates.

Nine virulence genes (*ipaBCD*, *virA*, *sen*, *set1A*, *set1B*, *ial*, *ipaH*, *stx* and *sat*) were detected (Figure 1). The *ipaH* gene was initially used to confirm the isolates as *Shigella*, and all the isolates (100%) were positive for this gene. The *ipaBCD* (85%) and *sat* (85%) were the most detected of the remaining genes, while *set1B* (18%) was the least detected. Three (9%) of the *S. flexneri* isolates harboured all the examined virulence genes investigated in the current study.

### 3.4. Clonal Relationships

**Figure 2:**
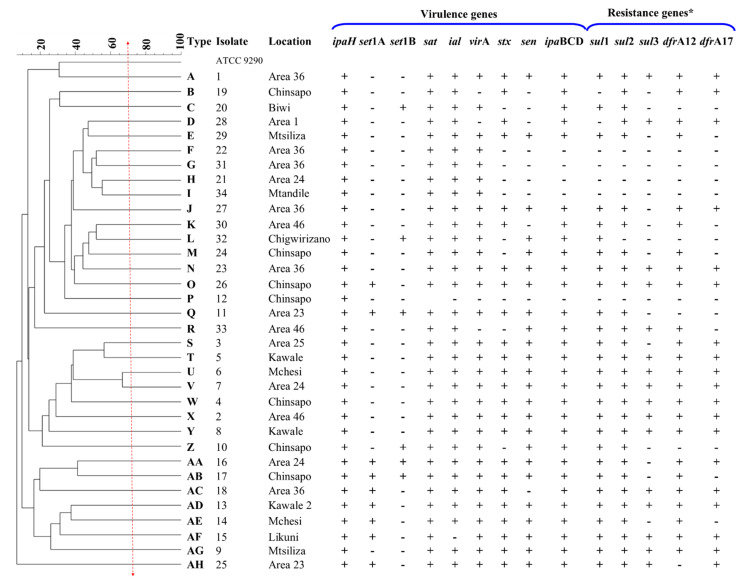
Enterobacterial-Repetitive-Polymerase Chain Reaction (ERIC-PCR) profiles of 34 *Shigella flexneri* isolates with annotated virulence and resistance genes. *Only genes that were detected at least once are reported.

The clonality analysis in this study showed that all the *S. flexneri* isolates were genetically distinct and clonally unrelated, irrespective of the location (Figure 2). ERIC-PCR analysis clustered the 34 isolates into 34 ERIC types designated A-AH using a 70.0% similarity cut-off. For instance, all seven *Shigella flexneri* isolates from Chinsapo were assigned to different ERIC types: B, M, O, P, W, Z and AB (Figure 2).

## 4. Discussion

Shigellosis is a public health problem among people living in low-income countries, especially in children, among whom it is considered an important cause of death [31]. We investigated 243 cases of acute bloody diarrhoea in OPD patients at Bwaila Hospital, Lilongwe, Malawi. *Shigella flexneri* was isolated from 34 patients (14%), and like other *Shigella* species that cause bloody diarrhoea, this species is associated with factors like poor hygiene and deficiency of drinking water [34]. Thus, the highest number of positive samples were recorded in Chinsapo and Area 36, two locations known to have poor sanitation environments, which may have contributed to this high level of shigellosis.

No other species was isolated from stool samples apart from *Shigella flexneri*, which is like that reported in eight African countries [35]. Similar trends have earlier been noted in Iran [36] and Ethiopia [37], where they reported 49.8% and 14.0%, respectively, of *Shigella flexneri* isolates. However, in a study conducted in Xinjiang, Uygur in China, although *Shigella flexneri* was the most prevalent species (79%), other species like *Shigella sonnei* were also reported [38]. The differences in species recorded in different studies is attributed to the geographical stratification of *Shigella* species due to countriess’ economic statuses. It has been reported that *S. flexneri* is mostly associated with infection in developing countries, while developed nations have greater cases of *S. sonnei*-associated diarrhoea; *S. boydii* is rarely recorded out of Bangladesh and South-East Asia [39]. However, *S. flexneri* remains the most isolated *Shigella* spp. globally [4].

Different age and gender pattern have been reported worldwide regarding the distribution of *Shigella* infections. For example, a study in China reported a higher prevalence of *S. flexneri* in males than in females; however, *S. sonnei* showed a reverse trend [40]. However, in the present study, most isolates were retrieved from male patients in the age group 12–45 years (30/34, 88.2% of isolates), similar to reports by another study in Iran [41]. The affected age group and gender are important for the epidemiology of shigellosis infections and appeared to be associated with the level of personal hygiene [42]. It has been suggested that females appeared to maintain better personal hygiene than males, as reported by Mayavati. et al. [43]. A large proportion of the community members recruited in this study lived in slums, which may explain their susceptibility to these diarrhoeal infections, consistent with studies done in Kenya [44].

Acute diarrhoeal diseases like shigellosis are usually self-limiting. However, antibiotic therapy is advocated to limit both the clinical condition of the illness and reduce faecal excretion of the organism. The emergence of antibiotic resistance in *Shigella* infections complicates the therapeutic management of shigellosis [45]. *Shigella* isolates from Lilongwe, as in other developing countries [46], showed high rates of resistance to commonly prescribed drugs, with all isolates being fully resistant to ampicillin (100%) and sulfamethoxazole/trimethoprim (100%). Similarly, *Shigella* isolates from Ethiopia [47], and Nigeria also displayed 100% resistance rates to ampicillin and sulfamethoxazole/trimethoprim [48]. This resistance trend has also been reported in Guatemala and Iran [49,50]. Sulfamethoxazole resistance in *Shigella* isolates is attributed to the acquisition of *sul 1*, *sul 2* or *sul 3* genes, encoding for dihydropteroate synthase enzymes which confer resistance to sulfonamides [51]. Of the total 34 (100%) that showed phenotypic resistance to sulfamethoxazole, 15 (44%) harboured all three *sul* resistance genes. This is consistent with trends previously reported in China [52]. The frequency of *sul 1 was* (79%), *sul 2* (82%) and *sul 3* (20%) as the lowest, which is in accordance with other studies reported in Bangladesh [51]. The non-detection of the *oxa* genes in the current study suggest that the phenotypic resistance observed could be due to other ESBLs-associated genes not tested. Therefore, further studies involving advanced methods like whole-genome sequencing would be needed to elucidate the genetic mechanisms of resistance in the studied isolates.

Invasiveness is an important aspect of pathogenesis in *Shigella* infections [52]. *Shigella* species can harbour several virulence factors associated with invasion of the colonic epithelium, facilitating their dissemination [53]. All the virulence genes tested in the present study (*ipaH, ipaBCD*, *sat*, *ial virA*, *sen, stx, set1A* and *set1B* were detected in at least one of the isolates assayed at different rates (Figure 1). Among these, *ipaH* was present in all the isolates, similar to reports in Bangladesh [54] and Ethiopia [55]. The *ipaH* (invasion plasmid antigen H) gene, together with the *ial* (invasion-associated locus) gene, mediate the ability of the organisms to colonise, penetrate and modify hosts’ epithelial cells, favouring cell-to-cell transmission [31]. Like the *ipaH* and *ial* genes, the characterisation of the *ipaBCD* gene, found in 85% of the study’s isolates, reveals that this gene aids *Shigella* species to invade host cell [56]. The virulence-associated invasion genes in *Shigella* are part of the invasive plasmid antigen (ipa), and *Shigella* grown in standard culture methods loses the virulence plasmid at a relatively high rate [57]. This likely accounted for the absence of *ipaBCD* in some of the isolates in this study. Nevertheless, this is the first study done on the distribution of virulence genes in Malawi to the best of our knowledge.

Molecular typing is performed to understand the epidemiology of the bacteria [58]. The isolates were epidemiologically unrelated, although they had similar antibiotic resistance profiles. This finding corroborated a study by Lluque et al. [59] in Peru that showed a high degree of genetic diversity with 23 different genotypes in 31 *Shigella flexneri* isolates using the pulse-field gel electrophoresis (PFGE) technique. Furthermore, Kosek et al. [60] also reported a high degree of heterogeneity in *S. flexneri* and *S. sonnei* isolates, as did Sati et al. [4] in Latin America. This extremely diverse pattern suggest that different strains of the same species could be circulating within the communities. However, further studies that involve the use of more resolute typing schemes such as the multi-locus sequence typing (MLST) and whole-genome sequencing (WGS) would be needed to substantiate this claim of the genetic unrelatedness of the *S. flexneri* strains in the study.

## 5. Conclusions

Shigellosis may be attributed to *Shigella flexneri* in Lilongwe, Malawi. Our analysis revealed high rates of resistance to ampicillin, sulfamethoxazole/trimethoprim, which are commonly used for empirical treatment of diarrhoeal infections. The *sul* genes were implicated in sulfamethoxazole resistance, while *dfrA12* and *dfrA17* genes were responsible for trimethoprim resistance. *OXA-1* and *OXA-2* genes were not detected genotypically. Given the geographical stratification of *Shigella* infections, regular local monitoring of susceptibility patterns needs to be implemented to guide empiric therapy. Additionally, access to safe drinking water, proper use of latrines and the promotion of handwashing should be encouraged to reduce the incidence of shigellosis.

## Data Availability

All data is contained within this article.

## Supplementary Materials

The following are available online at https://www.mdpi.com/article/10.3390/tropicalmed6020063/s1, Table S1: Primer sequences for detection of ampicillin, sulfonamide and trimethoprim resistant genes in *Shigella* spp., Table S2: Primer sequences for detecting virulence genes in *Shigella* spp. isolates., Table S3. Detection of sul, *dfrA* and *OXA* genes in phenotypically resistant isolates.

## References

[1] Kotloff K.L., Platts-Mills J.A., Nasrin D., Roose A., Blackwelder W.C., Levine M.M. (2017). Global burden of diarrheal diseases among children in developing countries: Incidence, etiology, and insights from new molecular diagnostic techniques. Vaccine.

[2] Sansonetti P.J. (2006). Shigellosis: An old disease in new clothes?. PLoS Med..

[3] Ranjbar R., Farahani A. (2019). Shigella: Antibiotic-resistance mechanisms and new horizons for treatment. Infect. Drug Resist..

[4] Sati H.F., Bruinsma N., Galas M., Hsieh J., Sanhueza A., Ramon Pardo P., Espinal M.A. (2019). Characterizing *Shigella* species distribution and antimicrobial susceptibility to ciprofloxacin and nalidixic acid in Latin America between 2000–2015. PLoS ONE.

[5] Morales-López S., Yepes J.A., Prada-Herrera J.C., Torres-Jiménez A. (2019). Enterobacteria in the 21st century: A review focused on taxonomic changes. J. Infect. Dev. Ctries..

[6] Barrantes K., Achí R. (2016). The importance of integrons for development and propagation of resistance in *Shigella*: The case of Latin America. Braz. J. Microbiol..

[7] Nave H.H., Mansouri S., Sadeghi A., Moradi M. (2016). Molecular diagnosis and anti-microbial resistance patterns among *Shigella* spp. isolated from patients with diarrhea. Gastroenterol. Hepatol. Bed Bench.

[8] Hosseini Nave H., Mansouri S., Emaneini M., Moradi M. (2016). Distribution of genes encoding virulence factors and molecular analysis of *Shigella* spp. isolated from patients with diarrhea in Kerman, Iran. Microb. Pathog..

[9] Omona S., Malinga G.M., Opoke R., Openy G., Opiro R. (2020). Prevalence of diarrhoea and associated risk factors among children under five years old in Pader District, northern Uganda. BMC Infect. Dis..

[10] Baker S., The H.C. (2018). Recent insights into *Shigella*: A major contributor to the global diarrhoeal disease burden. Curr. Opin. Infect. Dis..

[11] Ministry of Health (2015). Malawi Standard Treatment Guidelines (MSTG).

[12] Hussen S., Mulatu G., Yohannes Kassa Z. (2019). Prevalence of *Shigella* species and its drug resistance pattern in Ethiopia: A systematic review and meta-analysis. Ann. Clin. Microbiol. Antimicrob..

[13] Shahsavan S., Owlia P., Rastegar Lari A., Bakhshi B., Nobakht M. (2017). Investigation of efflux-mediated tetracycline resistance in *Shigella* isolates using the inhibitor and real time polymerase chain reaction method. Iran. J. Pathol..

[14] Bhattacharya D., Bhattacharya H., Thamizhmani R., Sayi D.S., Reesu R., Anwesh M., Kartick C., Bharadwaj A.P., Singhania M., Sugunan A.P. (2014). Shigellosis in bay of Bengal Islands, India: Clinical and seasonal patterns, surveillance of antibiotic susceptibility patterns, and molecular characterization of multidrug-resistant *Shigella* strains isolated during a 6-year period from 2006 to 2011. Eur. J. Clin. Microbiol. Infect. Dis..

[15] Hooper D.C., Jacoby G.A. (2015). Mechanisms of drug resistance: Quinolone resistance. Ann. N. Y. Acad. Sci..

[16] Paterson D.L., Bonomo R.A. (2005). Extended-spectrum beta-lactamases: A clinical update. Clin. Microbiol. Rev..

[17] Evans B.A., Amyes S.G.B. (2014). OXA β-lactamases. Clin. Microbiol. Rev..

[18] Adelowo O.O., Helbig T., Knecht C., Reincke F., Mäusezahl I., Müller J.A. (2018). High abundances of class 1 integrase and sulfonamide resistance genes, and characterisation of class 1 integron gene cassettes in four urban wetlands in Nigeria. PLoS ONE.

[19] Hammerum A.M., Sandvang D., Andersen S.R., Seyfarth A.M., Porsbo L.J., Frimodt-Møller N., Heuer O.E. (2006). Detection of *sul1*, *sul2* and *sul3* in sulphonamide resistant *Escherichia coli* isolates obtained from healthy humans, pork and pigs in Denmark. Int. J. Food Microbiol..

[20] Perreten V., Boerlin P. (2003). A new sulfonamide resistance gene (sul3) in *Escherichia coli* is widespread in the pig population of Switzerland. Antimicrob. Agents Chemother..

[21] Von Seidlein L., Deok R.K., Ali M., Lee H., Wang X.Y., Vu D.T., Do G.C., Chaicumpa W., Agtini M.D., Hossain A. (2006). A multicentre study of Shigella diarrhoea in six Asian countries: Disease burden, clinical manifestations, and microbiology. PLoS Med..

[22] Williams P.C.M., Isaacs D., Berkley J.A. (2018). Antimicrobial resistance among children in sub-Saharan Africa. Lancet Infect. Dis..

[23] Mokhtari W., Nsaibia S., Majouri D., Ben Hassen A., Gharbi A., Aouni M. (2012). Detection and characterization of Shigella species isolated from food and human stool samples in Nabeul, Tunisia, by molecular methods and culture techniques. J. Appl. Microbiol..

[24] EUCAST (2017). Breakpoint Tables for Interpretation of MICs and Zone Diameters. http://www.eucast.org.

[25] Seol S.Y., Kim Y.T., Jeong Y.S., Oh J.Y., Kang H.Y., Moon D.C., Kim J., Lee Y.C., Cho D.T., Lee J.C. (2006). Molecular characterization of antimicrobial resistance in Shigella sonnei isolates in Korea. J. Med. Microbiol..

[26] Lombardo M.N., G-Dayanandan N., Wright D.L., Anderson A.C. (2016). Crystal structures of trimethoprim-resistant dfrA1 rationalize potent inhibition by propargyl-linked antifolates. ACS Infect. Dis..

[27] Thungapathra M., Amita, Sinha K.K., Chaudhuri S.R., Garg P., Ramamurthy T., Nair G.B., Ghosh A. (2002). Occurrence of antibiotic resistance gene cassettes aac(6′)-Ib, dfrA5, dfrA12, and ereA2 in class I integrons in non-O1, non-O139 *Vibrio cholerae* strains in India. Antimicrob. Agents Chemother..

[28] Al-Assil B., Mahfoud M., Hamzeh A.R. (2013). First report on class 1 integrons and Trimethoprim-resistance genes from dfrA group in uropathogenic *E. coli* (UPEC) from the Aleppo area in Syria. Mob. Genet. Elements.

[29] Ouellette M., Bissonnette L., Roy P.H. (1987). Precise insertion of antibiotic resistance determinants into Tn2l-like transposons: Nucleotide sequence of the OXA-1. Proc. Natl. Acad. Sci. USA.

[30] Chmelnitsky I., Carmeli Y., Leavitt A., Schwaber M.J., Navon-Venezia S. (2005). CTX-M-2 and a new CTX-M-39 enzyme are the major extended-spectrum beta-lactamases in multiple *Escherichia coli* clones isolated in Tel Aviv, Israel. Antimicrob. Agents Chemother..

[31] Yaghoubi S., Ranjbar R., Dallal M.M.S., Fard S.Y., Shirazi M.H., Mahmoudi M. (2017). Profiling of virulence-associated factors in *Shigella* species isolated from acute pediatric diarrheal samples in Tehran, Iran. Osong Public Health Res. Perspect..

[32] Avcioglu N.H., Bilkay I.S. (2016). Antibiotic resistance, multidrug resistance and enterobacterial repetitive intergenic consensus polymerase chain reaction profiles of clinically important *Klebsiella* species. Asian Biomed..

[33] Chukwu M.O., Abia A.L.K., Ubomba-Jaswa E., Obi L.C., Dewar J.B. (2019). Antibiotic Resistance Profile and Clonality of E. coli Isolated from Water and Paediatric Stool Samples in the North-West, Province South Africa. J. Pure Appl. Microbiol..

[34] Nygren B.L., Schilling K.A., Blanton E.M., Silk B.J., Cole D.J., Mintz E.D. (2013). Foodborne outbreaks of shigellosis in the USA, 1998–2008. Epidemiol. Infect..

[35] Kahsay A.G., Muthupandian S. (2016). A review on Sero diversity and antimicrobial resistance patterns of *Shigella* species in Africa, Asia and South America, 2001–2014. BMC Res. Notes.

[36] Khaghani S., Shamsizadeh A., Nikfar R., Hesami A. (2014). *Shigella flexneri*: A three-year antimicrobial resistance monitoring of isolates in a Children Hospital, Ahvaz, Iran. Iran. J. Microbiol..

[37] Mulu W., Abera B., Yimer M., Hailu T., Ayele H., Abate D. (2017). Bacterial agents and antibiotic resistance profiles of infections from different sites that occurred among patients at Debre Markos Referral Hospital, Ethiopia: A cross-sectional study. BMC Res. Notes.

[38] Liu H., Zhu B., Qiu S., Xia Y., Liang B., Yang C., Dong N., Li Y., Xiang Y., Wang S. (2018). Dominant serotype distribution and antimicrobial resistance profile of *Shigella* spp. in Xinjiang, China. PLoS ONE.

[39] Anderson M., Sansonetti P.J., Marteyn B.S. (2016). Shigella diversity and changing landscape: Insights for the twenty-first century. Front. Cell. Infect. Microbiol..

[40] Mao Y., Cui E., Bao C., Liu Z., Chen S., Zhang J., Wang H., Zhang C., Zou J., Klena J.D. (2013). Changing trends and serotype distribution of *Shigella* species in Beijing from 1994 to 2010. Gut Pathog..

[41] Moosavian M., Ghaderiyan G.H., Shahin M., Navidifar T. (2019). First investigation of the presence of SPATE genes in *Shigella* species isolated from children with diarrhea infection in Ahvaz, southwest Iran. Infect. Drug Resist..

[42] Winter S.C., Dreibelbis R., Dzombo M.N., Barchi F. (2019). A mixed-methods study of women’s sanitation utilization in informal settlements in Kenya. PLoS ONE.

[43] Mhaske M.S., Pandve H.T., Kevin F., Khismatrao D.S., Kundap R.P. (2013). Morbidity pattern and personal hygiene in children among private primary school in urban area: Are the trends changing?. J. Fam. Med. Prim. Care.

[44] Kawakatsu Y., Tanaka J., Ogawa K., Ogendo K., Honda S. (2017). Community unit performance: Factors associated with childhood diarrhea and appropriate treatment in Nyanza Province, Kenya. BMC Public Health.

[45] Puzari M., Sharma M., Chetia P. (2018). Emergence of antibiotic resistant *Shigella* species: A matter of concern. J. Infect. Public Health.

[46] Surafel K., Geda K., Asefa K. (2015). Prevalence of *Shigella* related diarrhea in Ambo Town and antibiotic susceptibility of the isolated strains. Greener J. Epidemiol. Public Health.

[47] Belay A., Ashagrie M., Seyoum B., Alemu M., Tsegaye A. (2020). Prevalence of enteric pathogens, intestinal parasites and resistance profile of bacterial isolates among HIV infected and non-infected diarrheic patients in Dessie Town, Northeast Ethiopia. PLoS ONE.

[48] Pearson M., Chandler C. (2019). Knowing antimicrobial resistance in practice: A multi-country qualitative study with human and animal healthcare professionals. Glob. Health Action.

[49] Hegde S., Benoit S.R., Arvelo W., Lindblade K., López B., McCracken J.P., Bernart C., Roldan A., Bryan J.P. (2019). Burden of laboratory-confirmed shigellosis infections in Guatemala 2007–2012: Results from a population-based surveillance system. BMC Public Health.

[50] Avakh Majalan P., Hajizade A., Nazarian S., Pourmand M.R., Siyavoshani K.A. (2018). Investigating the prevalence of *Shigella* species and their antibiotic resistance pattern in children with acute diarrhea referred to selected hospitals in Tehran, Iran. J. Appl. Biotechnol. Rep..

[51] Iqbal M.S., Rahman M., Islam R., Banik A., Amin M.B., Akter F., Talukder K.A. (2014). Plasmid-mediated sulfamethoxazole resistance encoded by the *sul2* gene in the multidrug-resistant *Shigella flexneri* 2a isolated from patients with acute diarrhea in Dhaka, Bangladesh. PLoS ONE.

[52] Mattock E., Blocker A.J. (2017). How do the virulence factors of *Shigella* work together to cause disease?. Front. Cell. Infect. Microbiol..

[53] Kotloff K.L., Riddle M.S., Platts-Mills J.A., Pavlinac P., Zaidi A.K.M. (2018). Shigellosis. Lancet.

[54] Shahnaij M., Latif H.A., Azmi I.J., Amin M.B., Luna S.J., Islam M.A., Talukder K.A. (2018). Characterization of a serologically atypical *Shigella flexneri* Z isolated from diarrheal patients in Bangladesh and a proposed serological scheme for *Shigella flexneri*. PLoS ONE.

[55] Al-Maamory E.H., Al-Khafaji J.K., Al-Masoudi H.K. (2018). Detection the virulence-associated genes in *Shigella* species isolated from diarrheal samples in Babylon Province. J. Pharm. Sci. Res..

[56] Venkatesan M.M., Buysse J.M., Kopecko D.J. (1988). Characterization of invasion plasmid antigen genes (*ipaBCD*) from *Shigella flexneri*. Proc. Natl. Acad. Sci. USA.

[57] Pilla G., McVicker G., Tang C.M. (2017). Genetic plasticity of the Shigella virulence plasmid is mediated by intra- and inter-molecular events between insertion sequences. PLoS Genet..

[58] Ruppitsch W. (2016). Molecular typing of bacteria for epidemiological surveillance and outbreak investigation/Molekulare Typisierung von Bakterien für die epidemiologische Überwachung und Ausbruchsabklärung. Die Bodenkult. J. Land Manag. Food Environ..

[59] Lluque A., Mosquito S., Gomes C., Riveros M., Durand D., Tilley D.H., Bernal M., Prada A., Ochoa T.J., Ruiz J. (2015). Virulence factors and mechanisms of antimicrobial resistance in *Shigella* strains from periurban areas of Lima (Peru). Int. J. Med. Microbiol..

[60] Olortegui M.P., Hall E., Yori P.P., Gilman R.H., Burga R., Sanchez G.M., Bao J.P., Kosek M., Chavez C.B., Calderon M. (2012). Facilitated molecular typing of *Shigella* isolates using ERIC-PCR. Am. J. Trop. Med. Hyg..

