# Supplementary material for: Burden, Antibiotic Resistance, and Clonality of Shigella spp. Implicated in Community-Acquired Acute Diarrhoea in Lilongwe, Malawi
Source: Trop Med Infect Dis. 2021 Apr 28;6(2):63. doi: 10.3390/tropicalmed6020063 (PMC8167763; doi:10.3390/tropicalmed6020063)
Supplement: Supplementary file 1 [file tropicalmed-06-00063-s001.zip › tropicalmed-1160642-supplementary.pdf]

Tropical Medicine and Infectious Disease

MDPI

Article

Burden, antibiotic resistance, and clonality of *Shigella flexneri* implicated in community-acquired acute diarrhoea in Lilongwe, Malawi

Abel F.N.D. Phiri [1,2], Akebe Luther King Abia [1], Daniel Gyamfi Amoako [1], Rajab Mkakosya [3], Sabiha Y. Essack [1] and Gunnar Skov Simonsen [4,5*]

Tropical Medicine and Infectious Disease

MDPI

Table S1. Primer sequences for detection of ampicillin, sulfonamide and trimethoprim resistant genes in *Shigella* spp.

**Gene target**	**Primer**	**Sequence (5′ to 3′)**	**Annealing Temp (°C)**	**Reference**
OXA-1 group	*OXA-1F*	ACACAATACATATCAACTTCGC	55	Ouellette & Bissonnette, 1987
	OXA-1R	AGTGTGTTTAGAATGGTGATC		
OXA-2 group	*OXA-2F*	ATGGCAATCCGAATCTTCG	55	Chmelnitsky et al., 2005
	OXA-2R	TTATCGCGCAGCGTCCGAG		
dfrA1	*dfrA1F*	ACGGATCCTGGCTGTTGGTTGGACGC	58	Lombardo et al., 2016
	dfrA1R	CGGAATTCACCTTCCGGCTCGATGTC		
dfrA12	*dfrA12F*	GTTGCGGTCCAGACATAC	58	Thungpathra et al., 2002
	dfrA12R	CCGCCACCAGACACTA		
dfrA17	*dfrA17F*	TCGAGCTTCATGCCATTT	58	Al-Assil, 2013
	dfrA17R	TCTTCCATGCCATTCTGC		
sul1	*sul1F*	CTTCGATGAGAGCCGGCGGC	*	Ruiz, 2002
	sul1R	GCAAGGCGGAAACCCGCGCC		
sul2	*sul2F*	TCGTCAACATAACCTCGGACAG	*	Byne-Bailey, 2009
	sul2R	GTTGCGTTTGATACCGGCAC		
sul3	*sul3F*	GAGCAAGATTTTTGGAATCG	*	Perreten, 2003
	sul3R	CATCTGCAGCTAACCTAGGGCTTTGGA		

* Annealing temperatures were optimised at 62 °C as reported in the manuscript

Table S2. Primer sequences for detecting virulence genes in *Shigella* spp. isolates.

Gene target	Primer	Sequence (5′ to 3′)	Product size (bp)	Tm (°C)
Set 1 A	*ShET-1A*	F: TCACGCTACCATCAAAGA<br>R: TATCCCCCTTTGGTGGTA	309	55
Set 1B	*ShET-1B*	F: GTGAACCTGCTGCCGATATC<br>R: ATTTGTGGATAAAAATGACG	147	55
sat	*Sat1*	F: ACTGGCGGACTCATGCTGT<br>R: AACCCTGTAAGAAGACTGAGC	387	55
Ial	*Ial1*	F; CTGGATGGTATGGTGAGG<br>R; GGAGGCCAACAATTATTTCC	320	58
ipaH	*Shig1*	F: TGGAAAAACTCAGTGCCTCT<br>R: CCAGTCCGTAAATTCATTCT	423	58
virA	*virA*	F: CTGCATTCTGGCAATCTCTTCACATC<br>R-TGATGAGCTAACTTCGTAAGCCCTCC	215	58
Stx	*Stx1*	F: CAGTTAATGTGGTTGCGAAG<br>R: CTGCTAATAGTTCTGCGCATC	895	60
Sen	*ShET2*	F: ATGTGCCTGCTATTATTTAT<br>R: CATAATAATAAGCGGTCAGC	799	60
ipaBCD	*ipaBCD*	F: GCTATAGCAGTGACATGG<br>R: ACGAGTTCGAAGCACTC	612	60

Source: Yaghoubi, et al., 2017

Table S3. Detection of sul, dfrA and OXA genes in phenotypically resistant isolates.

Isolate number	Location	Resistance genes							
		sul1	*sul2*	*sul3*	*dfrA1*	*dfrA12*	*dfrA17*	*OXA-1*	*OXA-2*
1	Area 36	+	+	+	-	+	+	-	-
2	Area 46	+	+	+	-	+	+	-	-
3	Area 25	+	+	-	-	+	+	-	-
4	Chinsapo	+	+	+	-	+	+	-	-
5	Kawale	+	+	+	-	+	+	-	-
6	Mchesi	+	+	+	-	+	+	-	-
7	Area 24	+	+	+	-	+	+	-	-
8	Kawale	+	+	+	-	+	+	-	-
9	Mtsiliza	+	+	+	-	+	+	-	-
10	Chinsapo	+	+	-	-	-	-	-	-
11	Area 23	+	+	-	-	-	-	-	-
12	Chinsapo	-	-	-	-	-	-	-	-
13	Kawale 2	+	+	+	-	+	+	-	-
14	Mchesi	+	+	-	-	+	-	-	-
15	Likuni	+	+	+	-	+	+	-	-
16	Area 24	+	+	-	-	+	+	-	-
17	Chinsapo	+	+	-	-	+	-	-	-
18	Area 36	+	+	+	-	+	+	-	-
19	Chinsapo	-	+	-	-	+	+	-	-
20	Biwi	+	+	-	-	-	-	-	-
21	Area 24	-	-	-	-	-	-	-	-
22	Area 36	-	-	-	-	-	-	-	-
23	Area 36	+	+	+	-	+	+	-	-
24	Chinsapo	+	+	-	-	+	-	-	-
25	Area 23	+	+	+	-	-	+	-	-
26	Chinsapo	+	+	+	-	+	+	-	-
27	Area 36	+	+	-	-	+	+	-	-
28	Area 1	-	+	+	-	+	+	-	-
29	Mtsiliza	+	+	-	-	+	-	-	-
30	Area 46	+	+	-	-	+	-	-	-
31	Area 36	-	-	-	-	-	-	-	-
32	Chigwirizano	+	-	-	-	-	-	-	-
33	Area 46	+	+	+	-	+	-	-	-
34	Mtandile	-	-	-	-	-	-	-	-
	**Total**	**27**	**28**	**7**	**0**	**24**	**19**	**0**	**0**

+ = gene detected, - = gene not detected
